# The *Amomum tsao-ko* Essential Oils Inhibited Inflammation and Apoptosis through p38/JNK MAPK Signaling Pathway and Alleviated Gentamicin-Induced Acute Kidney Injury

**DOI:** 10.3390/molecules27207121

**Published:** 2022-10-21

**Authors:** Xiu-Jun Xu, Mei-Ling Zhang, Yan-Min Hou, Ke Zhang, Da-Hong Yao, Guo-Yu Li, Wei-Bing Kou, Hang-Yu Wang, Jin-Hui Wang

**Affiliations:** 1 Key Laboratory of Xinjiang Phytomedicine Resource and Utilization, Ministry of Education, College of Pharmacy, Shihezi University, Shihezi 832002, China; 2 Shihezi Institute for Drug Control, Shihezi 832002, China; 3 School of Pharmaceutical Sciences, Shenzhen Technology University, Shenzhen 518060, China; 4 Shenzhen Honghui Biopharmaceutical Co., Ltd., Shenzhen 518000, China; 5 State-Province Key Laboratory of Biomedicine-Pharmaceutics of China, Department of Medicinal Chemistry and Natural Medicine Chemistry, Harbin Medical University, Harbin 150081, China

**Keywords:** *Amomum tsao-ko*, essential oils, gentamicin, acute kidney injury, MAPK

## Abstract

The clinical application of gentamicin may lead to acute kidney injury (AKI), and the nephrotoxicity of gentamicin is related to the pathological mechanism of several oxidative and inflammatory cytokines. Plant-derived essential oils have good anti-inflammatory and antioxidant properties. This study aimed to clarify the protective effect of *Amomum tsao-ko* essential oils (AOs) on gentamicin-induced AKI in rats and its possible mechanism. The rat AKI model was induced by intraperitoneal injection of gentamicin. After 14 days of oral AO treatment, the renal function and pathological changes of the kidney tissues were evaluated, and the level of kidney tissue oxidative stress was detected. The content of inflammatory cytokines was measured by ELISA. The expression of ERK1/2, JNK1/2, p38, NF-κB, caspase-3, and Bax/Bcl-2 proteins were estimated by Western blot analysis. The results showed that taking AO reduced the contents of serum urea and creatinine in AKI rats and improve the pathological changes and oxidative stress of the kidney tissue in rats. At the same time, AO reduced inflammation and apoptosis during AKI by regulating the MAPK pathway. The data show that AO has a protective effect on the kidneys and may be a potential drug for treating kidney injury.

## 1. Introduction

Acute kidney injury is a clinical syndrome characterized by the rapid loss of renal function, which can further develop into chronic renal damage and even end-stage renal disease (ESRD) [1]. Gentamicin (GM) is used clinically to treat infections caused by Gram-negative bacteria. Because its clinical application may lead to AKI, its application in clinical trials is limited [2]. GM can be freely filtered in the glomerulus and reabsorbed in the proximal kidney tubules. Drugs selectively accumulate in the kidney’s proximal convoluted tubules, resulting in the loss of brush body integrity and kidney tubular obstruction followed by decreased glomerular filtration rate (GFR), thereby causing proteinuria and renal function damage [3,4,5]. GM nephrotoxicity is related to the pathological mechanisms of several oxidative and inflammatory cytokines, such as the production of free radicals, a reduction in antioxidant defense mechanisms, and acute tubular necrosis and glomerular congestion [6]. Despite some progress in improving the therapeutic effects for AKI, the current clinical treatment measures are still limited to the use of supportive treatment and dialysis. Unfortunately, the clinical use of multiple therapeutic drugs leads to kidney function damage and injury, and patients need to delay the start of supportive measures until their kidney function improves [7,8,9]. Therefore, new therapeutic methods and drugs need to be developed to improve the survival outcomes of AKI patients.

*Amomum tsao-ko* Crevost et Lemaire is the dry and mature fruit of cardamom in the ginger family, which is widely distributed in Southwest China. Oriental traditional medicine uses Amomum tsao-ko to treat malaria, throat infections, abdominal pain, stomach diseases, dyspepsia, nausea, vomiting, and diarrhea [10]. Monoterpenoids are the most abundant components in *Amomum tsao-ko* essential oils (AOs). To date, a series of studies have proven that monoterpenoids have anti-inflammatory, antibacterial, antiviral, hypoglycemic, immune regulation, antioxidant, anti-aging, and other biological activities [11,12]. Studies have shown that 1,8-cineole, the main component of the essential oils, has analgesic and/or anti-inflammatory properties and can treat diabetic nephropathy and inhibit podocyte damage [13,14]. Geraniol and citral also play a role in the treatment of drug-induced renal injury and glomerulosclerosis [15,16]. AO may also be an effective candidate drug for the treatment of AKI and is worthy of further development and research. Therefore, the purpose of this study was to determine the therapeutic effect of AO on gentamicin-induced AKI. At the same time, understanding its specific potential molecular mechanism is another important task.

## 2. Results

### 2.1. Determination Results of Chemical Components of the Amomum Tsao-Ko Essential Oils

**Figure 1:**
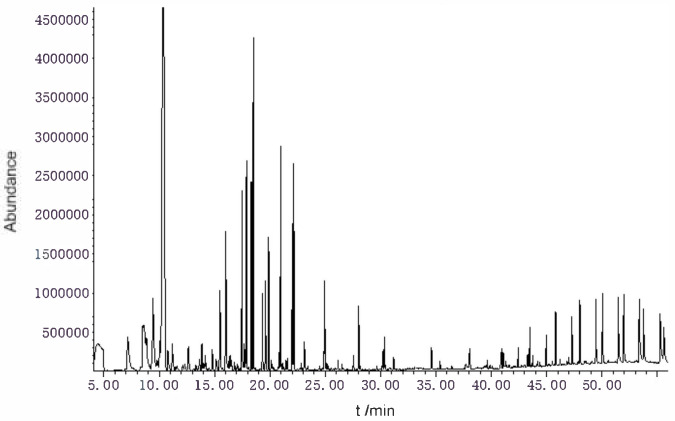
TIC diagram of *Amomum tsao-ko* essential oils.

- The *Amomum tsao-ko* essential oils were analyzed by gas chromatography–mass spectrometry (GC–MS). A total of 56 components were identified by GC–MS. the main components were eucalyptol (46.42%), (E)-citral (5.14%), 2,3-dihydro-1H-inden-e-4-carboxaldehyde (3.72%), geraniol (3.68%), geranyl acetate (3.60%) α-terpineol (3.48%), α-phellandrene (3.45%), 2-cyclohexen-1-ol (3.03%), (Z)-citral (3.00%), and β-methylcinnamylaldehyde (2.38%) (Table A1 and Figure 1).

### 2.2. Detection of GM-Induced Acute Kidney Injury in Rats

- Treatment of rats with gentamicin for 7 days resulted in a significant deterioration of kidney function. The contents of serum urea nitrogen (BUN) and serum creatinine (SCR) in GM-injected rats were significantly higher than those in the control group (*p* < 0.01). The enzyme activity of β-N-acetyl-glucosaminidase (NAG) increased significantly (*p* < 0.01), and the content of urinary protein and the lysozyme activity in the urine also increased significantly, indicating that rats’ renal filtration function and renal tubular reabsorption function were impaired after 7 days of continuous injection of GM. This showed that we successfully established an AKI model in rats (Table 1).

### 2.3. AO Improved the Pathological Changes in Kidney Tissue Induced by GM

**Figure 2:**
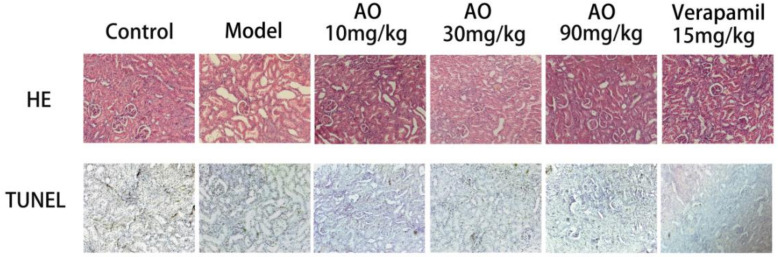
Effect of AO on histopathological changes of kidneys in GM-treated rats. Representative micrographs of each experimental group stained with hematoxylin and eosin and TUNEL, magnification ×200.

- The histopathological changes of rat kidney sections were examined. The normal rats showed normal glomerular and tubular structures. In the kidney sections of GM-treated rats, some kidney tubular epithelial cells were highly swollen and exhibited hydropic degeneration with severe vacuolization of the renal tubules. Most kidney tubular epithelial cells lost their brush edges. Exfoliated epithelial cells could be seen in the kidney tubules, the lumen was blocked, and a large number of inflammatory cells infiltrated the kidney interstitium. In AKI rats treated with AO and verapamil, the pathological damage of the renal tissue was improved in varying degrees, mainly by water-like degeneration of the renal tubular epithelial cells, necrosis of renal tubular epithelial cells, and the disappearance of inflammatory cell infiltration. TUNEL staining was used to detect apoptotic cells in renal tissue. Normal cells were stained blue, and apoptotic cells were stained yellow or brown. TUNEL-positive cells in the model group were mainly distributed in the renal cortex and renal tubules. After treatment, the number of apoptotic cells decreased gradually. The results showed that AO could reduce renal pathological injury and apoptosis of renal tubular epithelial cells and significantly improve renal tissue lesions in AKI rats (Figure 2).

### 2.4. AO Can Improve the Kidney Function of AKI Rats

**Figure 3:**
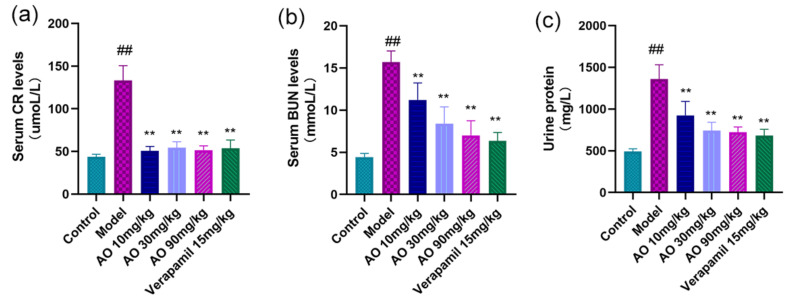
Effects of AO on kidney function indexes in AKI rats. (**a**) Serum creatinine, (**b**) blood urea nitrogen, and (**c**) urinary protein were measured on the first day after the last administration. The data are expressed as means ± standard deviation; compared with the model group, ** *p* < 0.01; compared with the control group, ## *p* < 0.01 (*n* = 6).

- AKI rats received AO and verapamil for 14 days, and compared with the normal control group, the contents of urinary protein and serum BUN and SCR in the model group were significantly higher (*p* < 0.01); Compared with the model group, the contents of urinary protein, BUN and SCR in the serum of rats in the different AO dosage groups and the verapamil group were significantly reduced (*p* < 0.01)(Figure 3).

### 2.5. AO Can Reduce the Oxidative Stress of Rat Kidney Induced by GM

**Figure 4:**
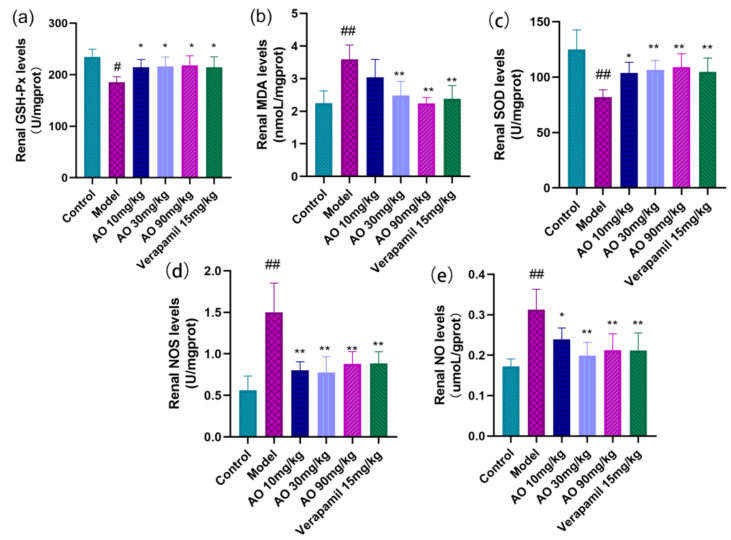
Regulation of AO on markers of oxidative tissue damage in kidney tissue of AKI rats. (**a**) Glutathione peroxidase (GSH-Ps), (**b**) malondialdehyde (MDA), (**c**) superoxide dismutase (SOD), (**d**) nitric oxide synthase (NOS), (**e**) nitric oxide (NO); # *p* < 0.05 vs. control group, ## *p* < 0.01 vs. control group. * *p* < 0.05,** *p* < 0.01 vs. model group (*n* = 6).

In order to distinguish whether the effect of AO on GM-induced AKI was related to antioxidant activity, we further measured some oxidation- and antioxidation-related indexes, including NO, NOS, MDA, SOD, and GSH-Px (Figure 4). The results showed that the contents of MDA, NOS, and NO in the kidney tissues of AKI rats induced by gentamicin increased significantly, and the activities of SOD and GSH-Px decreased significantly. After AO administration, the contents of MDA, NO, and NOS activity in the kidney tissue of AKI rats decreased significantly, and the activities of SOD and GSH-Px increased significantly. AO can regulate the oxidative antioxidant balance in the kidney tissue of AKI rats, which can help reduce GM-induced AKI.

### 2.6. AO Reduced the Levels of Inflammatory Cytokines in AKI Rats

**Figure 5:**
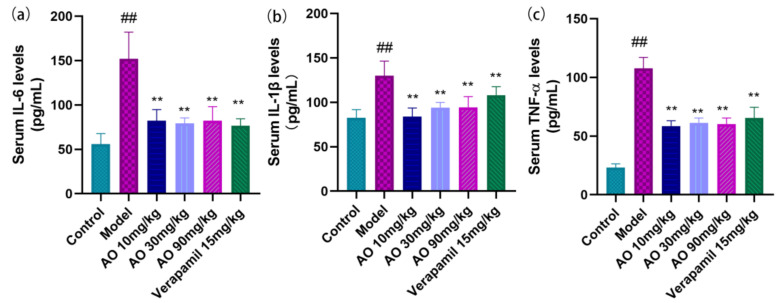
Effect of AO on the level of serum inflammatory factors in GM-treated rats. (**a**) IL-6 (interleukin 6), (**b**) IL-1β (interleukin -1β), and (**c**) TNF-α (tumor necrosis factor-α); *n* = 6; ## *p* < 0.01 vs. control group. ** *p* < 0.01 vs. model group.

- In addition to inducing oxidative tissue damage, AKI induced by GM was associated with the inflammatory response. GM significantly increased the inflammatory cytokines IL-6, IL-1β, and TNF-α in the serum (*p* < 0.01). After AO administration, the level of IL-6, IL-1β, and TNF-α decreased significantly (Figure 5).

### 2.7. Effect of AO on MAPK Pathway in AKI Rats

**Figure 6:**
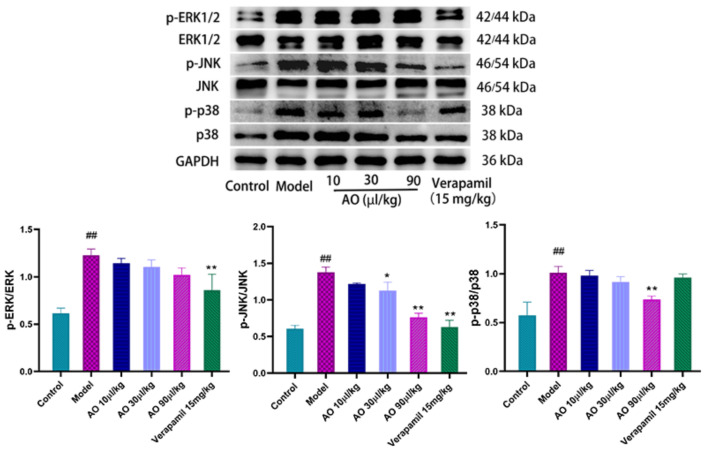
Effect of AO on the expression of MAPK pathway-related proteins in GM-induced AKI kidney of rats. ## *p* < 0.01 vs. control group. * *p* < 0.05, ** *p* < 0.01 vs. Model group.

- Mitogen-activated protein kinases (MAPKs) have been shown to play a key role in mediating and improving cellular stress responses, including exogenous toxicity. We evaluated the effect of the activation (phosphorylation) of MAPKs (ERK1/2, JNK1/2, and p38) on GM-induced AKI. The results showed that gentamicin stimulated the phosphorylation of the p38 and JNK1/2 proteins in the injured kidney, and the phosphorylation expression of the p38 and JNK1/2 proteins in the model group was significantly higher than that in the control group. By contrast, AO inhibited the phosphorylation of the p38 and JNK1/2 proteins in a dose-dependent manner. The above data show that the improvement of GM-induced AKI by AO is related to the phosphorylation of p38 and JNK1/2(Figure 6).

### 2.8. Effect of AO on the Expression of Bax, Bcl-2, Caspase-3, and NF-κB p65 in Kidney Tissue

An important pathological feature often observed in AKI is that the renal tubules are blocked by fragments of dead tubular epithelial cells, and then the surviving epithelial cells proliferate in these structures [17,18]. Cell death in the kidneys is caused by apoptosis and necrosis. The Bcl-2 and Bax proteins are important regulatory proteins of the apoptosis pathway, and they participate in the regulation of apoptosis through the mitochondrial pathway. Bax protein can activate the downstream apoptosis executive protein caspase-3 by affecting the mitochondrial pathway of apoptosis to promote apoptosis. Bcl-2 protein can combine with Bax protein to form a dimer and inhibit the expression of caspase-3 by affecting the flow of Ca2+ in cells. The results showed that AO downregulated the expression of the pro-apoptotic factor Bax and upregulated the expression of the anti-apoptotic protein Bcl-2, and also decreased the activation of caspase-3, which inhibited the apoptosis of renal tissue induced by gentamicin.

**Figure 7:**
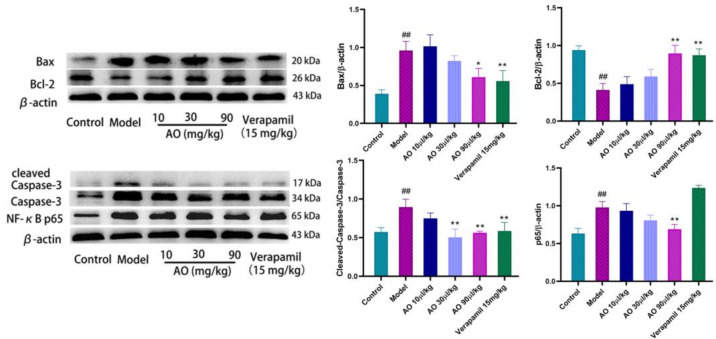
Effect of AO on the expression of proteins related to apoptosis and inflammation in kidneys of AKI rats induced by GM. ## *p* < 0.01 vs. control group. * *p* < 0.05 ,** *p* < 0.01 vs. model group.

As a crucial nuclear transcription factor, NF-κB has a wide range of biological activities. It can promote the transcription of chemokine genes, adhesion molecules, and various cytokines, and it plays an important role in the inflammatory response. The results of this experiment show that after AO administration, the expression of the NF-κB protein was inhibited in the process of AKI, suggesting that AO may inhibit the secretion of inflammatory factors through the NF-κB pathway (Figure 7).

## 3. Discussion

The main side effect of gentamicin is AKI, which limits its clinical application [2]. The main manifestations of gentamicin-induced AKI are a decrease in GFR, an increase in serum creatinine and blood urea nitrogen levels, and increased urinary protein excretion [4]. Our results showed that the levels of serum urea and creatinine in rats increased significantly after 7 days of GM injection, and the protein content and urinary enzyme level in urine increased significantly, which is consistent with AKI [16]. In AKI rats, oral AO for 14 days significantly restored most of the study parameters. In many previous studies, the lower glomerular filtration rate (GFR) after GM administration was the reason for the significant increase in urea and creatinine [19,20]. Some studies have shown that renal tubular casting caused by renal tubular epithelial cell abscission and brush edge abscission may help reduce GFR [17]. After GM injection, a large number of exfoliated tubular epithelial cells could be seen in the lumen of the rats’ renal tubules. After the oral administration of AO, the histopathological and renal function changes in the kidneys were improved. Therefore, AO may improve renal histopathology and ultimately reduce serum urea and creatinine levels.

It has been confirmed that inflammatory response plays a major role in the pathogenesis of GM-induced AKI. GM can increase the inflammatory infiltration of macrophages and produce TNF-α, IL-6, and other inflammatory factors, which promote the development of kidney inflammation [21]. GM accumulates in kidney tubular epithelial cells and activates MAPK, thereby activating the p38 and JNK signaling pathways, resulting in the production of inflammatory and apoptotic factors. GM can also induce monocyte NF-κB p65 protein expression-induced inflammation [22]. Recent studies have found that monoterpenes, the main chemical components in *Amomum tsao-ko*, have potential as key chemical mediators (such as pro-inflammatory and anti-inflammatory cytokines) to reduce inflammatory processes and regulate inflammation [23,24]. The NF-κB and MAPK signaling pathways are important pathways of monoterpenoids in the anti-inflammatory effect. Monoterpenoids can regulate cytokine production, which seems to be the main pharmacological mechanism of these compounds in reducing the inflammatory response [15,25]. Consistent with most studies, this study found that AO reduced the contents of IL-6, IL-1β, and TNF-α in the serum; inhibited the p38/JNK MAPK pathway; and reduced the expression of NF-κB p65, which proved that AO can reduce the inflammatory response in AKI.

In addition, the dysfunction of the oxidation/antioxidant balance system may be one of the important factors of acute kidney tubular epithelial cell apoptosis and necrosis caused by GM [26]. Studies have shown that GM increases the production of ROS, including hydroxyl radicals and superoxide anion radicals. In the case of GM injury, the body cannot prevent oxidative damage, which ultimately leads to renal function damage, reduced glomerular filtration, and an accumulation of metabolic end products [27]. Waste accumulates in the blood due to reduced GFR. GM-induced oxidative stress not only produces ROS but also produces RNS nitric oxide by increasing the expression of inducible NO. A low level of NO induces vasodilation, while a high level of NO induces cytotoxicity. Especially under the condition of oxidative stress, NO reacts with superoxide anions to produce cytotoxic peroxynitrite [28,29,30,31]. Eucalyptol, the main component of Amomum tsao-ko essential oils, has various biological activities, such as its anti-inflammatory and antioxidant properties, which make it a potentially important drug [32]. Eucalyptol was reported to be an effective drug in reducing the mitochondrial membrane potential, ROS, and NO levels in a neurodegenerative disease model in vitro [33]. It reduces 2,3,7,8-tetrachlorodibenzo-p-dioxin-induced oxidative stress in rat livers [34] and improves cerulein-induced acute pancreatitis by regulating oxidative stress [35]. Eucalyptol attenuated cigarette smoke-induced acute lung inflammation and oxidative stress in mice [36]. It had anti-inflammatory effects in an inflammatory model caused by lipopolysaccharides or other stimuli [37,38,39]. According to similar results reported in the literature and experiments, eucalyptol is the main material basis of AO ability to reduce oxidative stress and inflammation in renal injury. In addition, in the JNK/p38 MAPK cascade, ROS regulates the activation of MAPK through the positive circuit [40,41]. Some Bcl-2 family proteins, including the pro-apoptotic group and anti-apoptotic group, are controlled by the JNK and p38 MAPK cascades at a transcriptional and/or post-transcriptional level [42].

**Figure 8:**
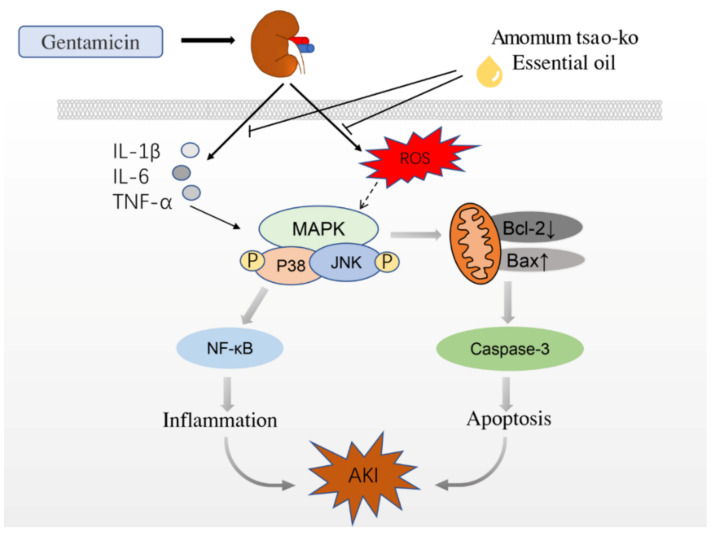
How AO affects the speculative mechanism of AKI caused by GM.

Stress-activated p38/JNK MAPKs play key roles in balancing cell survival and death in response to both extracellular and intracellular stresses. Samir A. Salama et al. found that GM could induce the phosphorylation of p38 MAPK and its downstream target gene c-fos [43]. Ahmed et al. established a rat AKI model by intraperitoneal injection of GM. They found that the expression levels of p38 MAPK, NF-κB p65, IL-1β, and TNF-α protein in rats with kidney injury were significantly higher than those in the control group [44]. T Ma et al. found that the expression of p38 MAPK and HSP27 protein increased 2 hours after AKI and decreased after the use of SB203580, a p38 MAPK protein inhibitor, indicating that AKI is related to p38 MAPK phosphorylation [45]. Most studies have shown that the pathogenesis of AKI is partly related to the activation of the p38 MAPK/JNK pathway in renal cells induced by stress. Blocking the activation of the p38 MAPK/JNK pathway can effectively inhibit the progress of inflammation and fibrosis in animal model nephropathy (Figure 8).

Bcl-2, an anti-apoptotic protein, stabilizes the mitochondrial membrane, while Bax, a pro-apoptotic protein, increases the permeability of the mitochondrial membrane [46]. P38 and JNK induce Bax release and translocation to the mitochondria, thereby promoting apoptosis. JNK can phosphorylate Bcl-2, thereby inhibiting its anti-apoptotic function [47,48]. In the process of GM-induced AKI, the increase in ROS plays a major role in the internal pathway of activating apoptosis through mitochondrial dysfunction [49]. In this pathway, the mitochondria-related proteins Bax and Bcl-2 are the main determinants. Maintaining the ratio of Bcl-2 to Bax can prevent apoptosis [42]; AO can significantly improve the level of active antioxidant species and help alleviate GM-induced oxidative stress. AO can regulate the oxidation/antioxidant balance and protect the kidneys. The results of this experiment show that AO can improve the oxidative antioxidant balance in the kidney tissues of AKI rats and reduce GM-induced AKI. At the same time, AO can reduce apoptosis in the process of renal injury by inducing the upregulation of Bcl-2, inhibiting the expression of Bax, and inhibiting the activation of the caspase-3 protein.

## 4. Materials and Methods

### 4.1. Materials

*Amomum tsao-ko* (Hebei yishengshan traditional Chinese Medicine Co., Ltd.); GM sulfate injection; verapamil (abbvie Deutschland GmbH & Co. KG, Düsseldorf, Germany); creatinine (Cr), urea nitrogen (BUN), a urine protein test kit, lysozyme (LZM), β-b-acetyl-glucosaminidase (NAG), nitric oxide (NO), nitric oxide synthase (NOS), superoxide dismutase (SOD), malondialdehyde (MDA), and total protein quantitative test kit (BCA) were purchased from Nanjing Jiancheng Bioengineering Institute (Nanjing, China); rat IL-6, rat IL-1β, and a rat TNF-α ELISA kit were purchased from Lianke Biology Co. Ltd., Hangzhou, China; P-SAPK/JNK antibody, p38/P-p38, NF-κB p65 were purchased from Cell Signaling Technology; ERK1/2 antibody, P-ERK1 + ERK2, caspase-3, Bax, Bcl-2, Goat anti-rabbit antibody, and Goat anti-mouse antibody were purchased from Wuhan bode Biology Co., Ltd., Wuhan, China; and GADPH TA-08 and β-Actin TA-09 were purchased from Beijing Zhongshan Jinqiao Biotechnology Co., Ltd., Shanghai, China.

### 4.2. Preparation of Essential Oil

The medicinal materials were crushed into a coarse powder and put into a flask; 5 times the amount of distilled water and broken porcelain chips were added, and the flask was connected to the essential oil tester and the reflux condenser. Water was added from the upper end of the condenser to fill the scale part of the essential oil tester and overflow into the flask. After soaking for 1 h, it was placed in an electric heating jacket, heated slowly until it boiled, and kept slightly boiling for about 5 h until the oil in the tester no longer increased, after which the heating was stopped. The essential oil was stored at 4 °C in the dark.

### 4.3. Determination of Essential Oil Composition

The compounds isolated from *Amomum tsao-ko* were analyzed by gas chromatography–mass spectrometry (GC–MS) using a 7890A/5975C gas chromatography–mass spectrometer (Agilent) and a DB-35MS (30 m × 0.25 mm, 0.25 μm) chromatographic column with a column flow rate 1.0 mL/min, a sample inlet temperature of 290 °C, a transmission line temperature of 250 °C, a four-stage rod temperature of 150 °C, and an ion source temperature of 230 °C in non-shunting mode. The temperature rise procedure was as follows: 50 °C was maintained for 1 min, the temperature was raised to 150 °C at 4 °C/min for 2 min, and then the temperature was raised to 280 °C at 5 °C/min for 2 min. The injection volume was 1 μL. Their mass spectra were further identified by comparing them with those stored in the NIST library. Relative percentages of the individual components of the essential oil were obtained by averaging the GC peak area% reports.

### 4.4. Animals and Experimental Design

Forty-eight male Sprague Dawley (SD) rats (200–250 g) were raised in each cage at a controlled temperature (23 ± 2 °C) and exposed to an artificial light/dark cycle of 12 h/12 h. Rats were allowed free access to food (granules) and water. The animal welfare and experimental procedures complied with the code of ethics for the care and use of experimental animals of the Experimental Ethical Inspection of First Affiliated Hospital, Shihezi University School of Medicine.

After 1 week of adaption to laboratory conditions, 8 rats in the blank control group were injected with normal saline every day, and the other rats were injected with GM (100 mg·kg^−1^·d^−1^) intraperitoneally every day for 7 days. After 7 days of injection, rats were transferred to the individual metabolic cage, 24 h urine and blood were collected for kidney function tests to judge whether the model was successfully established, and non-AKI rats were removed. AKI rats were randomly divided into a model group, AO groups with different doses (low, medium, and high), and a positive drug group (verapamil), with 6 rats in each group. From day 8, all rats were given 0.05 mL/10 g by gavage. The blank group and model group were given a 1% Tween-80 solution. Different doses of AO were given: 10 μL/kg, 30 μL/kg, and 90 μL/kg, respectively. The positive drug group was given verapamil (15 mg/kg) solution for 14 days. After the various initial treatments, animals in the different experimental groups were placed in metabolic cages, and 24 h urine samples were collected to determine urinary protein. Rats were euthanized after 1% pentobarbital anesthesia and blood loss. Blood samples were collected and centrifuged, and the serum was frozen until it was used for further analysis. The right kidney tissue was stored at −80 °C for further biochemical analysis.

### 4.5. Histology Examination

The left kidneys were fixed with 10% formalin for 24 h, dehydrated with graded ethanol, embedded in paraffin, sectioned at a thickness of 5 µm, and stained with hematoxylin and eosin to observe the pathological changes. TUNEL staining was used to detect the degree of apoptosis.

### 4.6. Kidney Function Assays

Serum creatinine, blood urea nitrogen, and urine protein were used as renal function markers; the contents of serum creatinine, blood urea nitrogen, and urine protein were determined by corresponding commercial kits, and all procedures were performed according to the manufacturer’s instructions.

### 4.7. Oxidative Stress Markers in Kidney Tissue

Kidney tissue was homogenized in cold saline to obtain 10% Kidney homogenate. The contents of NO and MDA and the activities of GSH-Px, SOD, and NOS were determined using the corresponding commercial kits according to the manufacturer’s protocol

### 4.8. Assessment of the Inflammatory Cytokines IL-1β, TNF-α, and IL-6

Different cytokine levels in the serum were determined using a commercial ELISA kit according to the manufacturer’s protocol, as previously described. Cytokine-specific antibodies pre-coated to microplates,, biotinylated antibody, and streptavidin-HRP were used in the experiment. TMB (3,3’, 5,5’-tetramethylbenzidine) reagent was used for detection. The optical density value was determined using a microplate reader and calculated at the linear portion of the curve.

### 4.9. Western Blot

The protein was separated by sodium dodecyl sulfate–polyacrylamide gel electrophoresis and transferred onto a polyvinylidene fluoride (PVDF) membrane. After blocking 5% skimmed milk with TBST, the membranes were incubated with primary antibodies against p38 (total and phosphorylated forms), JNK1/2 (total and phosphorylated forms), ERK1/2 (total and phosphorylated forms), NF-κB, caspase-3, Bcl-2, and Bax antigens at 4 °C overnight. After washing with TBST, it was incubated with a horseradish peroxidase-conjugated secondary antibody (1:10,000 dilution) at room temperature for 1 h. After washing 4 times with TBST, protein bands were formed on the membrane with enhanced chemiluminescence reagent. The gray value of the protein bands was detected by Image Pro Plus 6.0 software, and the ratio of β-actin or GAPDH was analyzed semi-quantitatively.

### 4.10. Statistical Analysis

Data were expressed as means ± S.D. of at least three independent experiments and evaluated using one-way analysis of variance (one-way ANOVA) followed by Bonferroni correction. *p* < 0.05 was considered statistically significant. The analyses were performed by SPSS 17.0.

## 5. Conclusions

In this study, AO reduced the levels of inflammatory factors and ROS induced by gentamicin and inhibited the activation of the p38/JNK MAPK pathway in rat kidneys. It alleviated the inflammatory reaction caused by NF-κB overexpression. At the same time, it also inhibited the apoptosis of renal tubular epithelial cells by regulating the expression of Bax, Bcl-2, and caspase-3. Our results demonstrate for the first time that AO plays a therapeutic role in GM-induced acute renal injury in rats by regulating the MAPK pathway and downstream anti-inflammatory and anti-apoptotic proteins. The new results of this study provide a pharmacological basis for further study of the application of AO in the treatment of nephropathy.

## Figures and Tables

**Table 1:** Effect of GM injection on kidney function in rats. (*n* = 6, X ± S).

Group	Urine Protein (mg/L)	BUN (mmoL/L)	SCR (μmoL/L)	NAG (U/L)	LZM (mg/L)
Control	554.60 ± 84.42	5.08 ± 0.99	40.99 ± 3.91	16.66 ± 5.73	0.29 ± 0.15
Gentamycin	1905.00 ± 145.20 ^##^	27.14 ± 3.14 ^##^	183.70 ± 27.95 ^##^	69.38 ± 17.01 ^##^	17.09 ± 1.21 ^##^

^##^*p* < 0.01 vs. control group.

## Data Availability

Data sharing not applicable.

## Sample Availability

Samples are available from the authors.

## Appendix A

**Table A1:** GC-MS analysis of The *Amomum tsao-ko* essential oils.

NO.	RT (min.)	Name	M.Wt	Peak Area %
1	7.15	(1S)-2,6,6-Trimethylbicyclo[3.1.1]hept-2-ene	136.13	2.03
2	8.52	.beta.-Pinene	136.13	1.01
3	8.86	7-Methyl-7H-dibenzo[b,g]carbazole	281.12	1.53
4	9.44	.alpha.-Phellandrene	136.13	3.45
5	9.79	(+)-4-Carene	136.13	0.53
6	10.05	p-Cymene	134.11	0.96
7	10.33	Eucalyptol	154.14	46.42
8	10.77	(Z)-3,7-Dimethyl-1,3,6-octatriene	136.13	0.74
9	11.19	.gamma.-Terpinene	136.13	0.85
10	12.13	1-Methyl-4-propan-2-ylidenecyclohexene	136.13	0.17
11	12.31	(2E,4E)-Deca-2,4-dienal	152.12	0.22
12	12.64	3,7-Dimethyl-1,6-octadien-3-ol	154.14	0.75
13	13.62	(4E,6Z)-2,6-Dimethylocta-2,4,6-triene	136.13	0.34
14	14.16	(5E)-3,3-Dimethylhepta-1,5-diene	124.13	0.40
15	14.80	Ethenylcyclohexane	110.11	0.46
16	15.14	L-.alpha.-Terpineol	154.14	0.43
17	15.49	(1R)-4-Methyl-1-propan-2-ylcyclohex-3-en-1-ol	154.14	2.17
18	16.02	.alpha.-Terpineol	154.14	3.48
19	16.18	4-Methyl-2,3-dihydrofuran	84.06	0.12
20	16.28	1,2,3,4,4a,8a-Hexahydronaphthalene	134.11	0.18
21	16.40	1,2-Diazaspiro(2.5)octane	112.10	0.34
22	16.54	(Z)-11-Tetradecen-1-yl acetate	254.23	0.24
23	16.79	N-Nitro-N’,N’-pentamethyleneguanidine	172.10	0.19
24	17.07	(3R)-3,7-Dimethyloct-6-en-1-ol	156.15	0.15
25	17.48	(2Z)-3,7-Dimethylocta-2,6-Dienal	152.12	3.00
26	17.59	cis-p-Mentha-2,8-dien-1-ol	152.12	0.10
27	17.89	Geraniol	154.14	3.68
28	18.08	1-Methyl-4-(1-Methylpropyl)-Benzene	148.13	0.12
29	18.32	2-Cyclohexen-1-ol	98.07	3.03
30	18.50	(E)-Citral	152.12	5.14
31	19.32	(1-Ethylpropyl)Benzene	148.13	1.21
32	19.88	Tetracyclo[3.3.1.0.1(3,9)]decan-10-one	148.09	2.18
33	20.12	trans-7-Ethyl-bicyclo[4.3.0]non-3-ene	150.14	0.20
34	20.21	2,4-Decadienal	152.12	0.10
35	20.98	2,3-Dihydro-1H-Indene-4-Carbaldehyde	146.07	3.72
36	21.13	2,3,4,5-Tetrahydro-1H-1,5-Benzodiazepine	148.10	0.14
37	21.46	N-Formyl-Dl-Phenylalanine	193.07	0.19
38	21.59	1,2-Dihydro-2-Methyl-3H-Indazol-3-One	148.06	0.21
39	22.03	(E)-Beta-Methylcinnamaldehyde	146.07	2.38
40	22.15	Geranyl acetate	196.15	3.60
41	22.84	Cyclopropanecarboxamidine	84.07	0.14
42	23.12	(E)-2-Decenyl acetate	198.16	0.46
43	23.43	2-Methoxy-1,3,4-Trimethylbenzene	150.10	0.14
44	25.08	trans-2-Dodecen-1-ol	184.18	0.12
45	26.16	2,5-Ditert-Butylphenol	206.17	0.19
46	26.53	(1S-cis)-4,7-Dimethy-1-(1-methylethyl)l-1,2,3,5,6,8a-Hexahydronaphthalene	204.19	0.11
47	27.55	2-[(1R,3S,4S)-4-Ethenyl-4-Methyl-3-prop-1-en-2-ylcyclohexyl]propan-2-ol	222.20	0.28
48	28.02	Nerolidol 2	222.20	1.14
49	31.18	2-[(4aS)-4a,8-dimethyl-2,3,4,5,6,7-hexahydro-1H-naphthalen-2-yl]propan-2-ol	222.20	0.25
50	35.38	Methyl trans-2-(3-cyclopropyl-7-norcaranyl)acetate	208.15	0.13
51	41.32	Z-8-Hexadecene	224.25	0.13
52	41.63	Heneicosane	296.34	0.11
53	43.28	6,8-Dimethyl-2-oxo-2H-chromene-4-carboxylic acid	218.06	0.26
54	44.19	1,8-Diazacyclotetradecane-2,9-dione	226.17	0.13
55	45.52	2-Ethylhexyl 3-(4-Methoxyphenyl)-2-Propenoate	290.19	0.10
56	47.01	2,2’-Methylenebis(4-methyl-6-tert-butylphenol)	340.24	0.13

## References

[1] Nagai J., Takano M. (2004). Molecular aspects of renal handling of aminoglycosides and strategies for preventing the nephrotoxicity. Drug Metab. Pharmacokinet..

[2] Balakumar P., Rohilla A., Thangathirupathi A. (2010). Gentamicin-induced nephrotoxicity: Do we have a promising therapeutic approach to blunt it?. Pharmacol. Res..

[3] Sassen M.C., Kim S.W., Kwon T.H., Knepper M.A., Miller R.T., Frøkiaer J., Nielsen S. (2006). Dysregulation of renal sodium transporters in gentamicin-treated rats. Kidney Int..

[4] Aronoff G.R., Pottratz S.T., Brier M.E., Walker N.E., Fineberg N.S., Glant M.D., Luft F.C. (1983). Aminoglycoside accumulation kinetics in rat renal parenchyma. Antimicrob. Agents Chemother..

[5] Schentag J.J., Gengo F.M., Plaut M.E., Danner D., Mangione A., Jusko W.J. (1979). Urinary casts as an indicator of renal tubular damage in patients receiving aminoglycosides. Antimicrob. Agents Chemother..

[6] Sahu B.D., Tatireddy S., Koneru M., Borkar R.M., Kumar J.M., Kuncha M., Srinivas R., ShyamSunder R., Sistla R. (2014). Naringin ameliorates gentamicin-induced nephrotoxicity and associated mitochondrial dysfunction, apoptosis and inflammation in rats: Possible mechanism of nephroprotection. Toxicol. Appl. Pharmacol..

[7] Ekor M., Emerole G.O., Farombi E.O. (2010). Phenolic extract of soybean (Glycine max) attenuates cisplatin-induced nephrotoxicity in rats. Food Chem. Toxicol..

[8] Lattanzio M.R., Kopyt N.P. (2009). Acute kidney injury: New concepts in definition, diagnosis, pathophysiology, and treatment. J. Am. Osteopat. Assoc..

[9] Rondon-Berrios H., Palevsky P.M. (2007). Treatment of acute kidney injury: An update on the management of renal replacement therapy. Curr. Opin. Nephrol. Hypertens..

[10] Wang Y., You C.X., Wang C.F., Yang K., Chen R., Zhang W.J., Du S.S., Geng Z.F., Deng Z.W. (2014). Chemical constituents and insecticidal activities of the essential oil from Amomum tsaoko against two stored-product insects. J. Oleo Sci..

[11] Quintans J.S.S., Shanmugam S., Heimfarth L., Araújo A.A.S., Almeida J.R.G.D.S., Picot L., Quintans-Júnior L.J. (2019). Monoterpenes modulating cytokines-A review. Food Chem. Toxicol..

[12] Cui Q., Wang L.T., Liu J.Z., Wang H.M., Guo N., Gu C.B., Fu Y.J. (2017). Rapid extraction of *Amomum tsao-ko* essential oil and determination of its chemical composition, antioxidant and antimicrobial activities. J. Chromatogr. B Analyt. Technol. Biomed. Life Sci..

[13] Cai Z.M., Peng J.Q., Chen Y., Tao L., Zhang Y.Y., Fu L.Y., Long Q.D., Shen X.C. (2021). 1,8-Cineole: A review of source, biological activities, and application. J. Asian Nat. Prod. Res..

[14] Kim D.Y., Kang M.K., Lee E.J., Kim Y.H., Oh H., Kang Y.H. (2018). Eucalyptol Inhibits Advanced Glycation End Products-Induced Disruption of Podocyte Slit Junctions by Suppressing Rage-Erk-C-Myc Signaling Pathway. Mol. Nutr. Food Res..

[15] Mahmoud N.M., Elshazly S.M., Rezq S. (2022). Geraniol protects against cyclosporine A-induced renal injury in rats: Role of Wnt/β-catenin and PPARγ signaling pathways. Life Sci..

[16] Yang S.M., Hua K.F., Lin Y.C., Chen A., Chang J.M., KuopingChao L., Ho C.L., Ka S.M. (2013). Citral is renoprotective for focal segmental glomerulosclerosis by inhibiting oxidative stress and apoptosis and activating Nrf2 pathway in mice. PLoS ONE.

[17] Bonventre J.V., Yang L. (2011). Cellular pathophysiology of ischemic acute kidney injury. J. Clin. Investig..

[18] Hosaka E.M., Santos O.F., Seguro A.C., Vattimo M.F. (2004). Effect of cyclooxygenase inhibitors on gentamicin-induced nephrotoxicity in rats. Braz. J. Med. Biol. Res..

[19] Laurent G., Kishore B.K., Tulkens P.M. (1990). Aminoglycoside-induced renal phospholipidosis and nephrotoxicity. Biochem. Pharmacol..

[20] Abd-Elhamid T.H., Elgamal D.A., Ali S.S., Ali F.E.M., Hassanein E.H.M., El-Shoura E.A.M., Hemeida R.A.M. (2018). Reno-protective effects of ursodeoxycholic acid against gentamicin-induced nephrotoxicity through modulation of NF-κB, eNOS and caspase-3 expressions. Cell Tissue Res..

[21] Han C., Sun T., Liu Y., Fan G., Zhang W., Liu C. (2020). Protective effect of Polygonatum sibiricum polysaccharides on gentamicin-induced acute kidney injury in rats via inhibiting p38 MAPK/ATF2 pathway. Int. J. Biol. Macromol..

[22] Kusaba T., Lalli M., Kramann R., Kobayashi A., Humphreys B.D. (2014). Differentiated kidney epithelial cells repair injured proximal tubule. Proc. Natl. Acad. Sci. USA.

[23] de Cássia da Silveira e Sá R., Andrade L.N., de Sousa D.P. (2013). A review on anti-inflammatory activity of monoterpenes. Molecules.

[24] Juergens U.R. (2014). Anti-inflammatory properties of the monoterpene 1.8-cineole: Current evidence for co-medication in inflammatory airway diseases. Drug Res..

[25] Beshay O.N., Ewees M.G., Abdel-Bakky M.S., Hafez S.M.N.A., Abdelrehim A.B., Bayoumi A.M.A. (2020). Resveratrol reduces gentamicin-induced EMT in the kidney via inhibition of reactive oxygen species and involving TGF-β/Smad pathway. Life Sci..

[26] Abdelrahman R.S. (2018). Protective effect of apocynin against gentamicin-induced nephrotoxicity in rats. Hum. Exp. Toxicol..

[27] Walker P.D., Shah S.V. (1988). Evidence suggesting a role for hydroxyl radical in gentamicin-induced acute renal failure in rats. J. Clin. Investig..

[28] Pedraza-Chaverrí J., Barrera D., Maldonado P.D., Chirino Y.I., Macías-Ruvalcaba N.A., Medina-Campos O.N., Castro L., Salcedo M.I. (2004). S-allylmercaptocysteine scavenges hydroxyl radical and singlet oxygen in vitro and attenuates gentamicin-induced oxidative and nitrosative stress and renal damage in vivo. BMC Clin. Pharmacol..

[29] Spiecker M., Darius H., Kaboth K., Hübner F., Liao J.K. (1998). Differential regulation of endothelial cell adhesion molecule expression by nitric oxide donors and antioxidants. J. Leukoc. Biol..

[30] Gong X., Celsi G., Carlsson K., Norgren S. (2012). Protective effects of N-acetylcysteine amide (NACA) on gentamicin-induced apoptosis in LLC-PK1 cells. Ren. Fail..

[31] Salama S.A., Arab H.H., Maghrabi I.A., Hassan M.H., AlSaeed M.S. (2016). Gamma-Glutamyl Cysteine Attenuates Tissue Damage and Enhances Tissue Regeneration in a rat Model of Lead-Induced Nephrotoxicity. Biol. Trace Elem. Res..

[32] Seol G.H., Kim K.Y. (2016). Eucalyptol and Its Role in Chronic Diseases. Adv. Exp. Med. Biol..

[33] Khan A., Vaibhav K., Javed H., Tabassum R., Ahmed M.E., Khan M.M., Khan M.B., Shrivastava P., Islam F., Siddiqui M.S. (2014). 1,8-cineole (eucalyptol) mitigates inflammation in amyloid Beta toxicated PC12 cells: Relevance to Alzheimer’s disease. Neurochem. Res..

[34] Ciftci O., Ozdemir I., Tanyildizi S., Yildiz S., Oguzturk H. (2011). Antioxidative effects of curcumin, β-myrcene and 1,8-cineole against 2,3,7,8-tetrachlorodibenzo-p-dioxin-induced oxidative stress in rats liver. Toxicol. Ind. Health.

[35] Lima P.R., de Melo T.S., Carvalho K.M., de Oliveira Í.B., Arruda B.R., de Castro Brito G.A., Rao V.S., Santos F.A. (2013). 1,8-cineole (eucalyptol) ameliorates cerulein-induced acute pancreatitis via modulation of cytokines, oxidative stress and NF-κB activity in mice. Life Sci..

[36] Kennedy-Feitosa E., Okuro R.T., Pinho Ribeiro V., Lanzetti M., Barroso M.V., Zin W.A., Porto L.C., Brito-Gitirana L., Valenca S.S. (2016). Eucalyptol attenuates cigarette smoke-induced acute lung inflammation and oxidative stress in the mouse. Pulm. Pharmacol. Ther..

[37] Kim K.Y., Lee H.S., Seol G.H. (2015). Eucalyptol suppresses matrix metalloproteinase-9 expression through an extracellular signal-regulated kinase-dependent nuclear factor-kappa B pathway to exert anti-inflammatory effects in an acute lung inflammation model. J. Pharm. Pharmacol..

[38] Zhao C., Sun J., Fang C., Tang F. (2014). 1,8-cineol attenuates LPS-induced acute pulmonary inflammation in mice. Inflammation.

[39] Santos F.A., Silva R.M., Campos A.R., De Araújo R.P., Lima Júnior R.C., Rao V.S. (2004). 1,8-cineole (eucalyptol), a monoterpene oxide attenuates the colonic damage in rats on acute TNBS-colitis. Food Chem. Toxicol..

[40] Zeng K.W., Song F.J., Wang Y.H., Li N., Yu Q., Liao L.X., Jiang Y., Tu P.F. (2014). Induction of hepatoma carcinoma cell apoptosis through activation of the JNK-nicotinamide adenine dinucleotide phosphate (NADPH) oxidase-ROS self-driven death signal circuit. Cancer Lett..

[41] Zhang X., Wang X., Wu T., Li B., Liu T., Wang R., Liu Q., Liu Z., Gong Y., Shao C. (2015). Isoliensinine induces apoptosis in triple-negative human breast cancer cells through ROS generation and p38 MAPK/JNK activation. Sci. Rep..

[42] Yue J., López J.M. (2020). Understanding MAPK Signaling Pathways in Apoptosis. Int. J. Mol. Sci..

[43] Salama S.A., Arab H.H., Maghrabi I.A. (2018). Troxerutin down-regulates KIM-1, modulates p38 MAPK signaling, and enhances renal regenerative capacity in a rat model of gentamycin-induced acute kidney injury. Food Funct..

[44] Ahmed H.I., Mohamed E.A. (2019). Candesartan and epigallocatechin-3-gallate ameliorate gentamicin-induced renal damage in rats through p38-MAPK and NF-κB pathways. J. Biochem. Mol. Toxicol..

[45] Ma T., Liu X.W., Liu Z. (2013). Function of the p38MAPK-HSP27 pathway in rat lung injury induced by acute ischemic kidney injury. Biomed. Res. Int..

[46] Fricker M., Tolkovsky A.M., Borutaite V., Coleman M., Brown G.C. (2018). Neuronal Cell Death. Physiol. Rev..

[47] Tsuruta F., Sunayama J., Mori Y., Hattori S., Shimizu S., Tsujimoto Y., Yoshioka K., Masuyama N., Gotoh Y. (2004). JNK promotes Bax translocation to mitochondria through phosphorylation of 14-3-3 proteins. EMBO J..

[48] Kim B.J., Ryu S.W., Song B.J. (2006). JNK- and p38 kinase-mediated phosphorylation of Bax leads to its activation and mitochondrial translocation and to apoptosis of human hepatoma HepG2 cells. J. Biol. Chem..

[49] El Gamal A.A., AlSaid M.S., Raish M., Al-Sohaibani M., Al-Massarani S.M., Ahmad A., Hefnawy M., Al-Yahya M., Basoudan O.A., Rafatullah S. (2014). Beetroot (Beta vulgaris L.) extract ameliorates gentamicin-induced nephrotoxicity associated oxidative stress, inflammation, and apoptosis in rodent model. Mediat. Inflamm..

